# Influence of Surface Discharge on Resin Degradation in Decay-like Fracture of Composite Insulators

**DOI:** 10.3390/polym15040790

**Published:** 2023-02-04

**Authors:** Qian Wang, Weining Bao, Yanfeng Gao, Shuqi Liu, Shuming Liu, Zhou Zuo, Chao Wu, Xidong Liang

**Affiliations:** 1 State Key Laboratory of Power System, Department of Electrical Engineering, Tsinghua University, Beijing 100084, China; 2 China Electric Power Planning and Engineering Institute, Beijing 100084, China; 3 State Grid Jibei Electric Power Co., Ltd. Research Institute, North China Electric Power Research Institute Co., Ltd., Beijing 100045, China

**Keywords:** composite insulator, fiber-reinforced polymer, abnormal fracture, surface discharge, resin degradation

## Abstract

Composite insulators have gradually become the preferred approach for electrical insulation in power systems, especially in polluted areas. Composite insulators consist of three main components: the shed, rod, and end fitting. Insulators withstand mechanical stresses via rods that are composed of glass-fiber-reinforced epoxy (GFRE). However, regardless of the high tensile strength of GFRE rods, in real-life operation, abnormal fractures have frequently been reported all over the world, which substantially increase the risk of major accidents in power systems. Fractural accidents mainly consist of brittle and decay-like fractures, which exhibit rather different morphologies at the cross sections. Brittle fracture has been effectively eliminated, while the mechanism of decay-like fracture has still not been clearly revealed. In this study, surface discharge tests were applied to investigate the discharge influence on the degradation of GFRE. The test successfully simulated the composition variation of the rods in real-life composite insulators with decay-like fractures. Moreover, it confirmed that the distinction between the characteristics of brittle fracture and decay-like fracture stems from epoxy degradation due to hydrolysis and carbonization. In addition, the respective influences of the resin type, glass fiber type, and acid liquid immersion on the degradation process were probed, and the degradation mechanism proposed in this research was verified. Based on the results, measures for preventing the development of decay-like fractures in real-life operations were determined.

## 1. Introduction

Overhead transmission lines have been, are, and are still likely to be the major approach to electric power delivery over long distances due to their large transmission capacities and low construction costs [1,2]. Insulators, which are an essential part of transmission towers, play a vital role, considering that they simultaneously assure both electrical and mechanical reliability [3,4]. In recent years, composite insulators (or polymeric insulators) have become more and more popular in comparison with traditional porcelain and glass insulators due to their excellent hydrophobicity, low weight, and convenience of maintenance [5,6]. For instance, over 10 million strings of composite insulators have already been used at the different levels of high-voltage transmission lines in China [7]. A composite insulator mainly consists of three components [8,9,10]. The metal end fittings at both ends of the insulator are for sealing and fixing. The shed and sheath, which are usually composed of silicone rubber composites, guarantee a sufficient surface flashover gradient and prevent the rod inside from being affected by environmental conditions. In the meantime, glass-fiber-reinforced epoxy (GFRE) is used to manufacture the core rod, which bears the mechanical load of electrical conductors. The tensile strength of GFRE composites is considerably high, which allows for a sufficient margin for stretching the load at the design stage [11,12].

**Figure 1:**
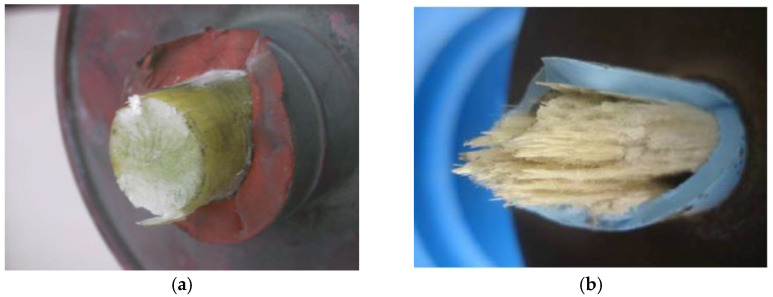
Typical cross-section images of composite insulators with abnormal fractures after real-life operation: (**a**) brittle fracture; (**b**) decay-like fracture.

However, electrical departments all over the world continuously report composite insulators that have experienced fracture accidents [13,14]. These accidents substantially increase the risks of falling conductors and the collapses of transmission towers, which are both considered to be major accidents for power systems. Thus, the investigation into the mechanisms behind these fractures has attracted massive attention from both engineers and researchers. The reported fractures tend to occur at the mechanical load, which is prominently lower than the rated load; thus, they cannot simply be referred to as mechanical failures and are instead referred to as abnormal fractures [15]. In general, the abnormal fractures of composite insulators comprise two types: brittle fracture and decay-like fracture, as shown in Figure 1 [16,17]. The former has been attributed to the stress corrosion of the glass fiber in GFRE [18]. To be specific, the partial discharge around transmission towers during real-life operation, such as corona, can lead to the ionization of the surrounding atmosphere and the generation of nitrogen oxides. Next, the nitrogen oxides gradually turn into nitric acid under wet conditions and permeate the silicone rubber sheath. The glass fiber (E fiber) in the GFRE then corrodes due to the nitric acid, which places it under mechanical stress, which then causes it to fracture [19]. More recently, the application of electrical-corrosion-resistant fiber (ECR fiber) effectively suppressed the occurrence of brittle fractures [20]. By contrast, decay-like fracture exhibits a clearly distinct cross-section, the mechanism of which has still not yet been revealed. In this paper, “brittle fractures” and “decay-like fractures” specifically refer to the fracture accidents that are found in real-life composite insulators. These fractures consist of the features of both fibers and polymers, as these are always used together. Thus, these fracture terms are not totally consistent with the terms that are used for material characterization. To be specific, here, a brittle fracture refers to a fracture accident with a flat cross-section in which the fiber is fractured while the polymer is hardly affected. In comparison, decay-like fracture denotes a fracture accident with a rugged cross-section in which the fiber is fractured and the polymer is degraded. Fiber fracture and polymer degradation are fundamentally different, and they should be separately described in most cases.

The importance of liquid permeation, and especially acid liquid, on the decay-like fractures of GFRE rods, has been recognized in previous research [21,22]. In addition, the “decay” characteristics shown in Figure 1b suggest the severe degradation of the resin matrix, which cannot be the result of liquid immersion only. Hence, considering the abnormal temperature increase detected in actual operation, overheating is most likely another cause [23,24]. Moreover, because the overheating area is highly concentrated, it should be attributed to the current concentration or partial discharge instead of the overall temperature increase in the insulator [25]. Hence, different kinds of surface discharge tests are designed and conducted to simulate the physic-chemical characteristics of actual composite insulators with decay-like fractures. However, the influences of the different influencing factors that are involved in the process of resin degradation have not been investigated. As a result, no effective preventive measures can be accordingly proposed to suppress the development of a decay-like fracture. Thus, in this study, the influences of the hydrolysis resistance of the resin, the composition of the glass fiber, and the immersion in nitric acid on the resin degradation in GFRE rods were probed in detail. Based on the results and analysis, we identified specific preventive measures for actual operation, including the preferred material selection of the glass fiber, resin matrix, and coupling agent.

## 2. Materials and Methods of Surface Discharge Test

### 2.1. Material and Experimental Setup

GFRE rods with diameters of 5 mm were used in this research, the formulation and manufacturing techniques of which were consistent with those for the rods of the composite insulators that operate in actual transmission lines. The rods were provided by Teporel Electrical Inc., and they were fabricated using the pultrusion technique. The diameter of the glass fiber in the GFRE was around 20 μm, and bisphenol A-type epoxy resin was selected as the polymer matrix. An experimental setup was prepared for the simulation of the surface discharge, which is minutely introduced in Ref. [24]. The GFRE sample was placed in a closed cavity with a pair of annular electrodes that were fixed at both ends of the rod. The distance between the electrodes was set to 100 mm, and the applied AC voltage was 10 kV (root mean square). In addition, to simulate the liquid immersion, which increases the surface conductivity of GFRE rods, an ultrasonic device was used to vaporize the 0.1 g/L of NaCl solution into the cavity.

### 2.2. Results of Surface Discharge Test and Discussion

**Figure 2:**
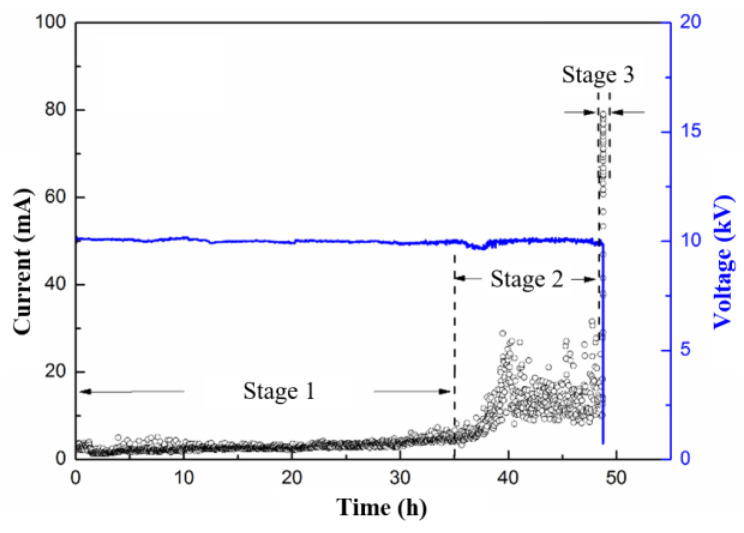
Amplitude variations of applied voltage (blue line) and measured current (black points) during surface discharge test of pristine GFRE rod.

The spectra of the applied voltage and measured current before the occurrence of flashover are shown in Figure 2. The current in the figure denotes the maximum value measured within each minute, which is also applicable to the related figures in the following text. Based on the results, three stages were recognized.

**Figure 3:**
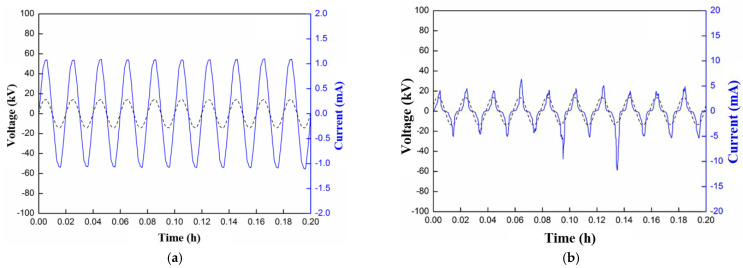
Waveforms of surface currents measured at Stages 1 and 2 of the surface discharge test: (**a**) Stage 1; (**b**) Stage 2.

Stage 1: GFRE hydrolysis. At the beginning of the test, the surface discharge is weak, as the sample surface is in good condition and without structural damage. The measured current spectra are almost perfectly sinusoidal with similar amplitudes (lower than 1 mA), as shown in Figure 3a. During this stage, surface discharge occasionally occurs, which originates from one of the electrodes and appears blue or purple in color. Consequently, the epoxy resin in the vicinity of the concentrated-discharge area is oxidized. The discharge is discontinuous, it is randomly distributed around the GFRE rod, and it leaves minute and white channels on the sample surface, which indicates the degradation of the epoxy resin. Then, the minute channels gradually develop, and they finally run through the entire area between the electrodes. Hence, a hydrolysis channel is preliminarily formed along the sample surface.

Stage 2: The development of a hydrolysis channel. After several tens of hours of Stage 1, the discharges in blue or purple gradually turn yellow, which indicates a higher intensity, as shown in Figure 2. Moreover, the discharge occurrence becomes more frequent, and there is a certain randomness in the amplitude, as suggested in Figure 3b. Moreover, the discharges are most concentrated in the areas adjacent to the hydrolysis channel formed in Stage 1, which results in the more severe degradation of the epoxy, as well as carbonization, to a certain degree.

Stage 3: Flashover. The resistance of the hydrolysis channel continuously decreases under wet conditions during Stage 2, which is due to the degradation of the epoxy resin and conductive fog, and which is confirmed by the prominent current increase in Figure 2. Then, the initiation of the surface arc and its march along the sample surface become feasible. The current spectrum becomes totally irregular. This eventually leads to the flashover between the electrodes and the termination of the surface discharge test.

### 2.3. Composition Variation of GFRE Rod after Surface Discharge Test

**Figure 4:**
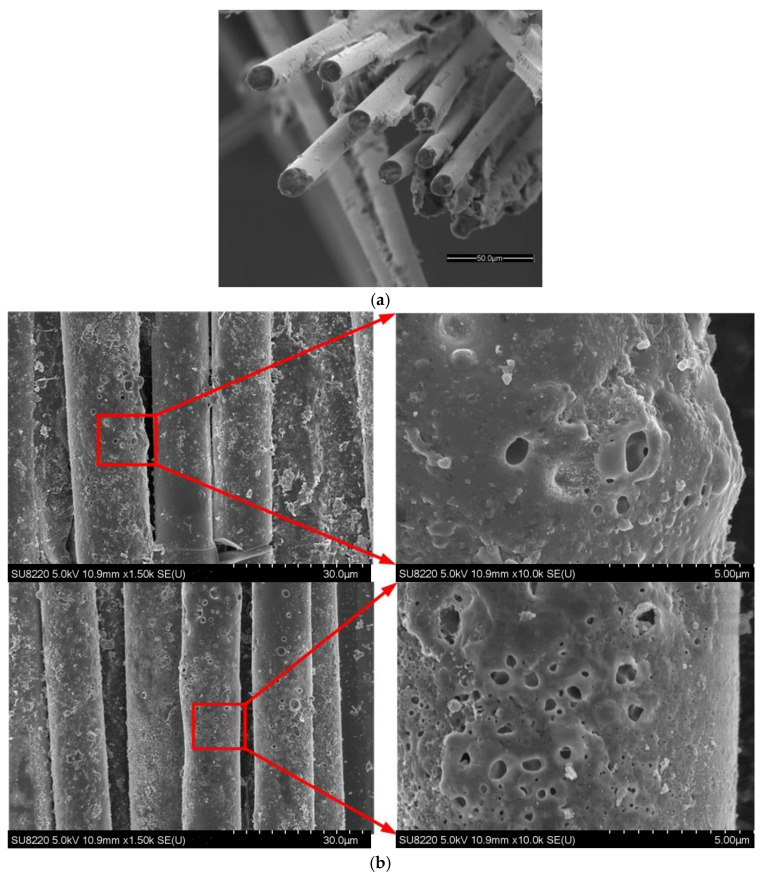
Micromorphologies of GFRE rods before (prepared by mechanical failure) and after surface discharge test, characterized by SEM: (**a**) before test; (**b**) after test.

To compare the micromorphologies of the GFRE rod before and after the surface discharge test, a GFRE rod with mere mechanical failure was prepared. The SEM images conducted by SU8220, Hitachi are shown in Figure 4, and they suggest that, compared with the pristine sample, most of the epoxy resin was ablated, and it disappeared after the surface discharge test. The remaining epoxy on the surface of the glass fiber became porous, the mechanism of which is discussed in detail in Ref. [26].

**Figure 5:**
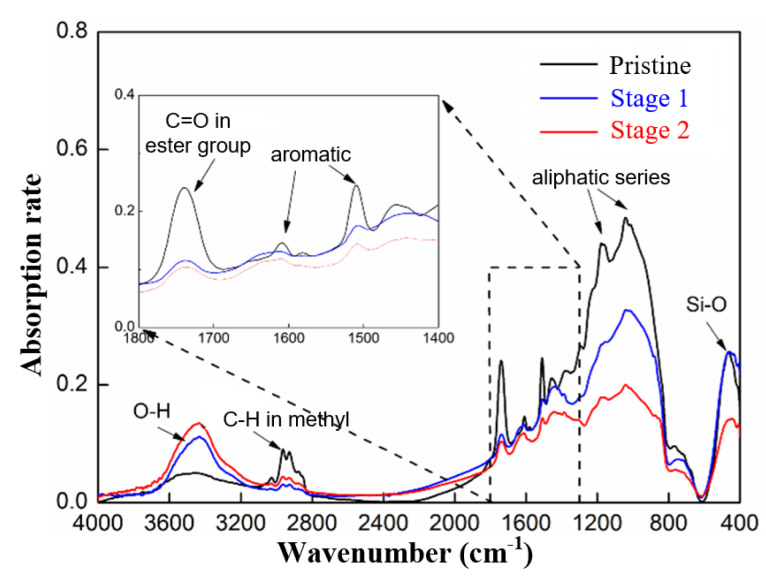
FTIR results of GFRE rod at different stages of surface discharge test.

**Figure 6:**
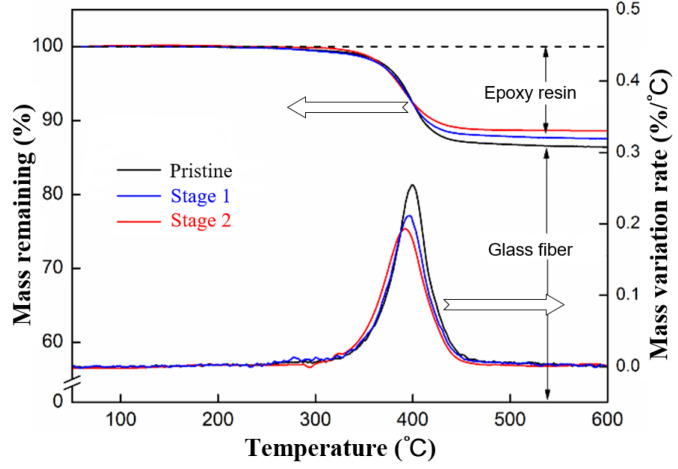
TGA results of GFRE rod at different stages of surface discharge test.

The composition variations at the different stages of the surface discharge test were investigated using FTIR (Fourier-transform infrared spectroscopy) and TGA (Thermos-gravimetric analysis), the results of which are shown in Figure 5 and Figure 6, respectively. The results indicate a decrease in the resin content in the GFRE sample, as well as decreases in the major functional groups in the epoxy resin, including the ester, aliphatic and aromatic compounds. By contrast, the C=O content substantially increased, which was the result of carbonization. These characteristics are all in accordance with actual composite insulators with a decay-like fracture [27]. The effectiveness of the surface discharge test for the simulation of the chemical reaction during the decay-like fracture process was confirmed. Additionally, the spectra of the leakage current could be used to evaluate the resistance of the rod sample to the development of a decay-like fracture.

**Figure 7:**
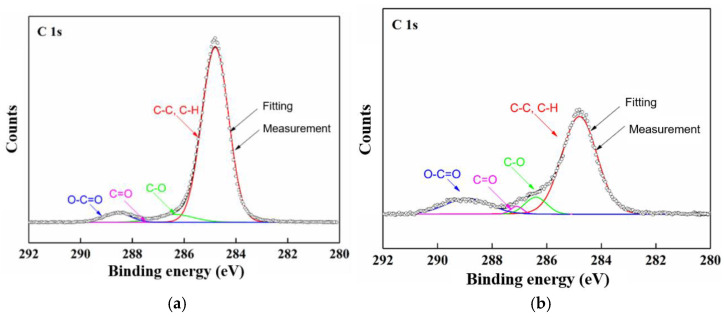

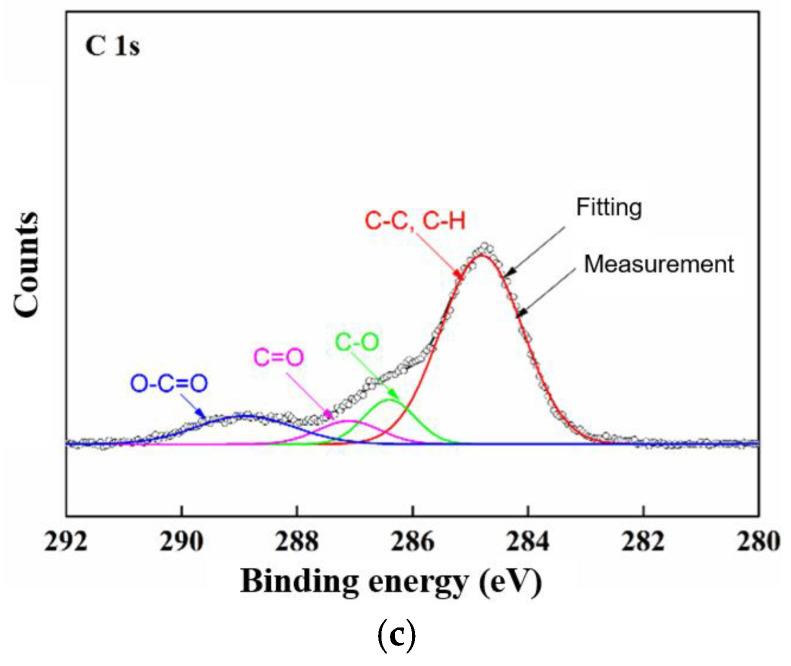
XPS results for C element of GFRE rod at different stages of surface discharge test: (**a**) pristine; (**b**) Stage 1; (**c**) Stage 2.

**Figure 8:**
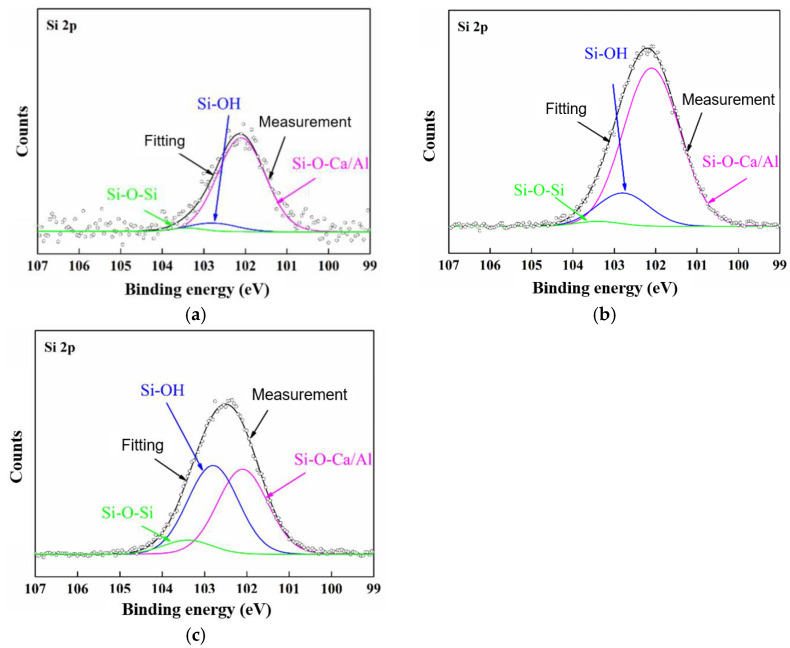
XPS results for Si element of GFRE rod at different stages of surface discharge test: (**a**) pristine; (**b**) Stage 1; (**c**) Stage 2.

Furthermore, XPS (X-ray photoelectron spectroscopy) was applied to probe the variation in the binding conditions of the C and Si elements, as shown in Figure 7 and Figure 8, respectively. According to the results, during Stage 1, the C element in the C–C and C–H decreased, while the C element in the C–O, C=O, and O–C=O, with higher oxidation states, increased, which was due to the oxidation effects, and especially in the hydrolysis channel. Then, in Stage 2, the acute discharge further accelerated the oxidation of the C. In comparison, the Si–O–Al/Ca structure was converted into Si–OH and Si–O–Si due to its exposure to acid, which resulted in the corresponding variation in the proportion of the functional groups.

## 3. Surface Discharge Tests under Different Conditions

### 3.1. Influence of Hydrolysis Resistance of Resin on Surface Discharge Test

**Figure 9:**
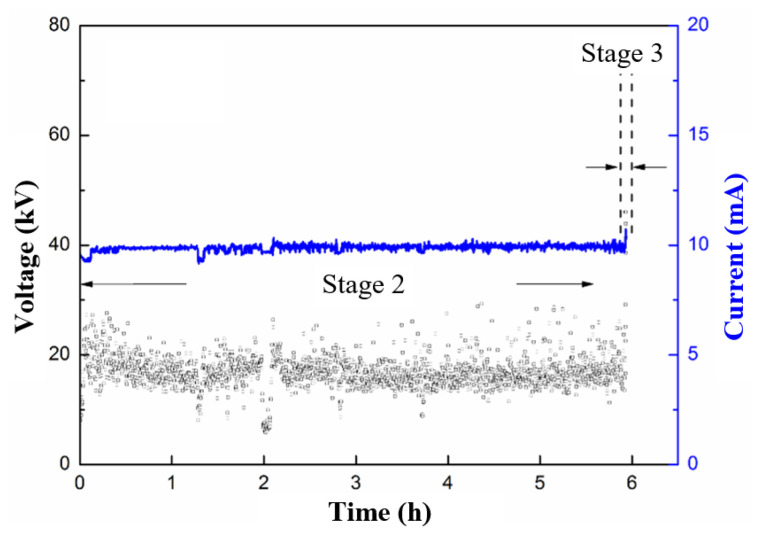
Amplitude variation of applied voltage (blue line) and measured current (black points) during surface discharge test of insulator rod made of polyester resin and glass fiber.

Apart from the epoxy resin in use currently, polyester resin is also considered to be one of the candidate matrixes for the rods of composite insulators, the hydrolysis resistance of which is weaker than that of epoxy resin [28]. Therefore, a special rod made of glass fiber and polyester resin was manufactured using the same pultrusion technique as was used for the GFRE rods. Additionally, a surface discharge test was conducted on the glass-fiber-reinforced polyester rod, and the results are shown in Figure 9.

Here, the duration of Stage I was short in comparison with that of the GFRE, and it could hardly be recognized. That is, the discharge on the rod surface was more liable to result in the degradation of the polyester. Therefore, the hydrolysis channel that connects the electrodes was formed within a shorter period of time. Additionally, with reference to Stage 2, due to the acceleration of the hydrolysis, the average current amplitude was much higher than that in Figure 2. Within only 6 h, the hydrolysis channel became conductive enough to trigger the flashover. In addition, the duration of Stage 3 was also short, which is indicated by the current surge at the end of the spectrum. According to the results, epoxy resin is preferable to polyester resin, and for composite insulators in actual operation, resin matrixes with stronger hydrolysis resistances should be mandatory. The surface discharge test used in this study can be applied as an effective evaluation method.

### 3.2. Influence of Immersion in Nitric Acid on Surface Discharge Test

**Figure 10:**
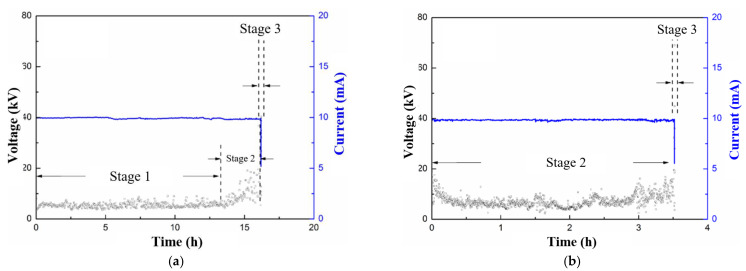
Amplitude variations of applied voltage (blue line) and measured current (black points) during surface discharge test of GFRE rod after immersion in 1 mol/L HNO_3_ for different durations: (**a**) 10,000 h; (**b**) 40,000 h.

Previous research has demonstrated that immersion in liquid, and especially acid liquid, results in the failure of the fiber–epoxy interface, which is also a key factor for the development of decay-like fracture [29]. Therefore, in this research, GFRE rods were immersed in nitric acid before the surface discharge test to probe the coupling effect of the acid immersion and surface discharge. The results of the leakage current of the GFRE rods after immersion are shown in Figure 10. The selection of the immersion time was based on the close relationship between the liquid permeation process and the square root of the time [30]. Moreover, the permeation process into the GFRE is rather slow, which is why at least 10,000 h is recommended. However, 40,000 h is over 4 years, which is difficult to extend further. In Figure 10a, the duration of Stage 1 is prominently shorter compared with that in Figure 2, while the average current amplitude is much higher. Likewise, Stage 2 in Figure 10a is also accelerated. The current amplitudes at Stage 1 and Stage 2 are similar here, and they are, thus, hard to distinguish clearly. In reference to Figure 10b, no obvious Stage 1 can be identified, which indicates that the rod surface had already been destroyed by nitric acid, and, thus, the connecting hydrolysis channel could be easily formed. Additionally, during Stage 2, the hydrolysis process was further enhanced, and the flashover was accomplished within 4 h of the beginning of the test. As a comparison, it took around 49 h for the flashover of the pristine GFRE rod, which illustrates the vital role of the fiber–resin interface in the hydrolysis process.

Thus, it is important to avoid the acid immersion of insulator rods in actual operation. To achieve this, a coupling agent (which is the adhesive between the sheath and rod) with better resistance to acid should be applied, which would prevent the acid molecules from penetrating through the silicone rubber sheath and further permeating the rod, thereby suppressing the development of decay-like fractures. The surface discharge test provides a quantitative approach to the evaluation of the corrosion degrees of actual composite insulators by acid liquids.

**Figure 11:**
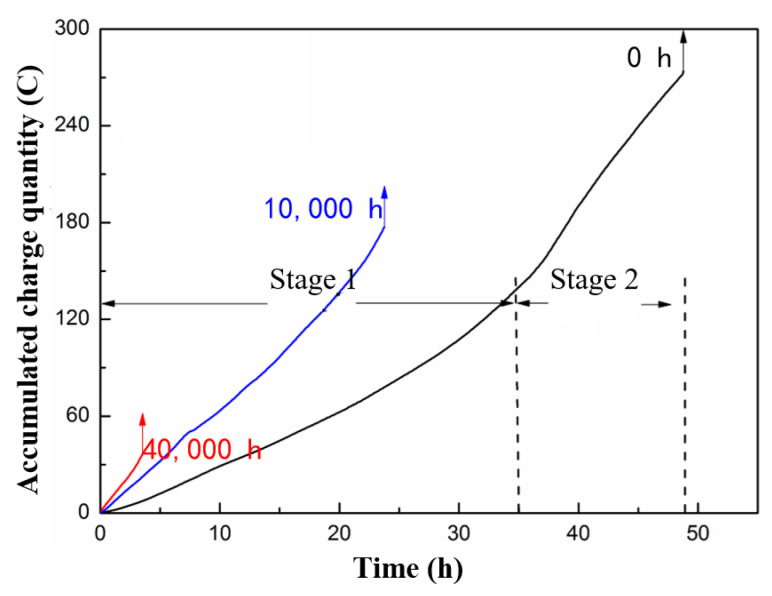
Variation in accumulated charge quantity during surface discharge test of GFRE rod after immersion in 1 mol/L HNO_3_ for different durations, calculated using MATLAB.

Given that the spectra of the current amplitude could only be used to qualitatively or semiquantitatively characterize the process, we needed to establish the accumulated charge quantity [31], which refers to the integral of the leakage current over time, the variation of which, during the test is shown in Figure 11, and which was numerically calculated using MATLAB. The slope of the spectrum denotes the leakage current according to its definition.

### 3.3. Influence of Glass Fiber on Surface Discharge Test

**Figure 12:**
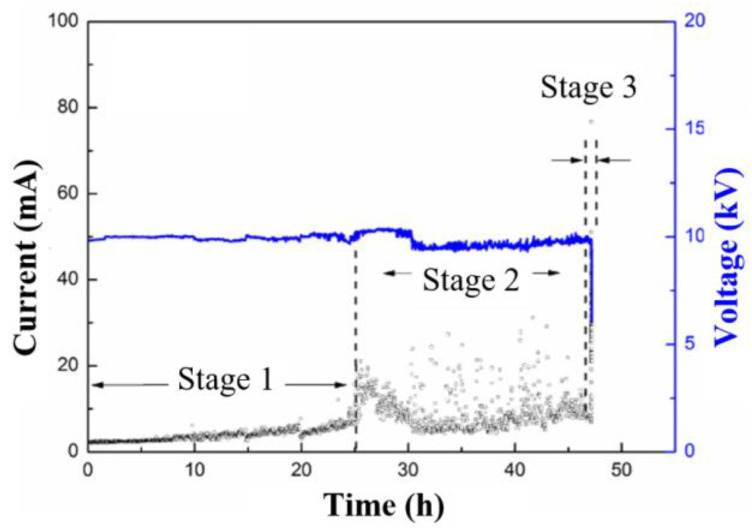
Amplitude variation of applied voltage (blue line) and measured current (black points) during surface discharge test of GFRE rod made from E fiber.

The brittle fracture of composite insulators has been effectively suppressed in actual operation by replacing the traditional E fiber in the GFRE with ECR fiber, which does not contain the B (boron) element. However, it seems that decay-like fractures cannot be simultaneously eliminated. Almost all accident insulators with decay-like fractures are made from ECR fiber. Thus, there is the question of whether decay-like fractures can only occur in GFRE rods with ECR fiber. A surface discharge test was conducted on a GFRE rod made from E fiber for comparison. Additionally, the rod was manufactured with the same pultrusion technique. The result of the surface discharge test is shown in Figure 12, and it indicates a similar trend and magnitude as shown in Figure 2. Stage 3 can hardly be recognized from the spectrum in Figure 12 owing to the short duration. However, Stage 3 denotes the flashover process, which, technically, is always existent. Thus, it is still listed in the figure.

According to the results in Figure 2 and Figure 12, the total times of the tests before the flashover were similar, which suggests that the surface discharge makes the same contribution to GRFE rods made from E fiber as those made from ECR fiber. Consequently, the resistance to the discharge is mainly determined by the epoxy resin in GFRE instead of the glass fiber. Therefore, an explanation can be accordingly derived for the abovementioned question. The development of decay-like fracture simultaneously requires acid–liquid immersion and partial discharge. Additionally, it takes a relatively long time, and typically several years, in actual operation. By contrast, the development of brittle fractures could be accomplished within a day. Thus, if the insulator rod is made from E fiber, after exposure to acid liquid, brittle fracture could occur within a short period of time, leaving no room for the development of a decay-like fracture. As a result, decay-like fracture is rarely found in composite insulators with E fiber, which suggests the excellent resistance of ECR fiber to nitric acid.

## 4. Discussion

**Figure 13:**
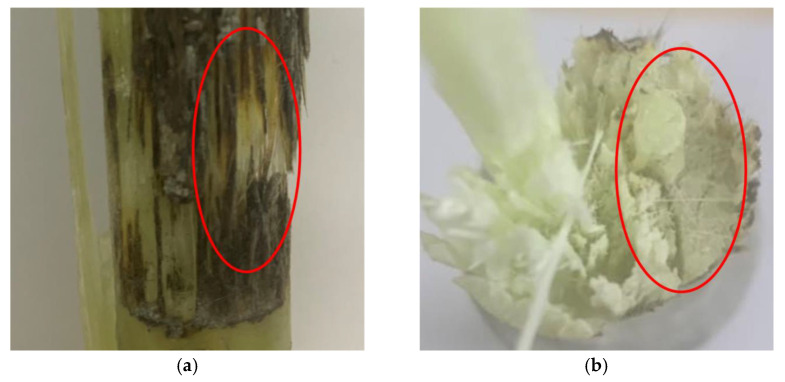
GFRE rod of 18 mm made of E fiber after surface discharge test and acid liquid immersion test, showing features of both brittle fracture and decay-like fracture. (**a**) Decay-like fracture; (**b**) Brittle fracture.

To draw a sharper comparison between the morphologies of brittle fracture and decay-like fracture, another test was designed to obtain the features of both kinds of fractures at the same cross-section. To be specific, a GFRE rod made of E fiber with a diameter of 18 mm was prepared. First, a surface discharge test was carried out with a tensile strength of 340 MPa. After 24 h, the test was manually terminated, which should take place at the end of Stage 1 or the beginning of Stage 2, according to Figure 12. Next, the area between the electrodes after the surface discharge test was put into 1 mol/L of HNO_3_. The rod was fractured within 12 h, and its cross-section is shown in Figure 13, in which the features of both brittle fracture and decay-like fracture can be recognized. The dark area in Figure 13a suggests the direct ablation of the resin (carbonization) due to strong discharge and after the conductivity of the hydrolysis channel became high enough. The flat area can be attributed to the brittle fracture of the rod, which originated from the stress corrosion of the E fiber, as mentioned above. Meanwhile, the epoxy in the flat area was hardly degraded, as the inner part of the rod was not affected during the surface discharge test. The rugged area resembles the decay-like fracture observed in operation, with its color becoming yellow and with the characteristics of carbonization.

To accomplish the test within a reasonable amount of time, the surface discharge test in this research was much more acute than those conducted under the actual environmental conditions of insulators. Surface discharge is rarely found in actual insulators unless the sheath is seriously damaged and the GFRE rod is totally exposed. In most cases, the current concentration is the origin of the degradation, and it could stem from defects in the raw material or from damage introduced during manufacturing. In view of a large number of insulators operating in the power system, as well as the accuracy of the measurement, monitoring the leakage current of each insulator is difficult. The detection of an abnormal temperature increase is more feasible, which nowadays can be conducted using infrared thermal imagers. By replacing the insulators with abnormal temperature increases, a substantial number of decay-like-fracture accidents could be eliminated in the early stage. In addition, the widespread application of unmanned aerial vehicles is more convenient for electrical departments.

## 5. Conclusions

In this research, surface discharge tests were applied to investigate the influence of the discharge on the development of a decay-like fracture. The results confirm the effectiveness of the test in simulating the composition variation of the GFRE during the fracture process. We derived several major conclusions, as follows.

Resin degradation is the main distinction between the cross-section morphologies of brittle fracture and decay-like fracture. Partial discharge and overheating are the origins of epoxy degradation.

Hydrolysis is a vital component of resin degradation. The flashover of a rod made from polyester (6 h) is much faster than that of a rod made from epoxy (49 h). Thus, resins with good resistance to hydrolysis should be used for the manufacturing of insulator rods.

Acid liquid immersion substantially accelerates the degradation process. The flashover can be accomplished within 4 h of the rod being sufficiently affected by nitric acid. Therefore, a coupling agent with good resistance to acid should be applied to prevent further liquid immersion into GFRE.

The type of glass fiber does not influence the degradation of the resin. However, brittle fracture occurs within short periods of time for E fiber after exposure to acid liquid, while the degradation of epoxy takes longer. Hence, decay-like fracture is only found in composite insulators made from ECR fiber.

The surface discharge test in this research is an efficient approach to the quantitative evaluation of the resistance of the rod material to decay-like fracture. However, although the physicochemical characteristics of decay-like fracture were simulated, the morphology of the cross-section still exhibited distinctions that are found in actual operation. Therefore, the setup of the experiment can be modified, for example, by decreasing the discharge intensity and extending the degradation process. Other new methods for simulating decay-like fracture could be proposed.

## Data Availability

Data will be made available upon reasonable request.
